# Anti-Collapsin Response Mediator Protein 5(CV2/CRMP5) and Anti-Glutamic Acid Decarboxylase (GAD) Antibodies-Mediated Encephalopathy Mimicking Atypical Parkinsonism

**DOI:** 10.3390/neurolint16060132

**Published:** 2024-12-16

**Authors:** Giuseppe Schirò, Matteo Gastaldi, Salvatore Iacono, Silvia Scaranzin, Valentina Picciolo, Valentina Arnao, Anita Ferrari, Cesare Gagliardo, Marco D’Amelio

**Affiliations:** 1 Department of Biomedicine, Neuroscience and Advanced Diagnostics (BiND), University of Palermo, 90127 Palermo, Italy; giuseppeschiro1994@gmail.com (G.S.); salvo.iak@gmail.com (S.I.); valentinapicciolo.vp@gmail.com (V.P.); anita.ferrari16@gmail.com (A.F.); cesare.gagliardo@unipa.it (C.G.); 2 Neuroimmunology Laboratory and Neuroimmunology Research Section, IRCCS Mondino Foundation, Via Mondino 2, 27100 Pavia, Italy; matteo.gastaldi@mondino.it (M.G.); silvia.scaranzin@mondino.it (S.S.); 3 UO Neurologia e Stroke Unit, Azienda di Rilievo Nazionale ad Alta Specializzazione, Ospedali Civico Di Cristina Benfratelli, 90134 Palermo, Italy; arnao.valentina@gmail.com

**Keywords:** atypical parkinsonism, anti-CV2/CRMP5 antibodies, anti-GAD antibodies, paraneoplastic neurological syndrome

## Abstract

**Background**: Paraneoplastic neurological syndromes (PNSs) are rare conditions characterized by immune-mediated pathogenesis, frequently associated with the presence of a neoplasm. Although a single antineuronal antibody mediates a specific syndrome, atypical manifestations mediated by the same antibody have been described. **Methods**: The aim of this study was to report on an atypical case of PNS with dual positivity for anti-GAD65 and anti-CRMP5/CV2 antibodies, simultaneously characterized by cognitive decline associated with progressive ataxia and parkinsonism. We also reviewed the current literature for published cases of PNSs with parkinsonism associated with anti-GAD65 and anti- CRMP5/CV2 antibodies. **Results**: A 68-year-old man with an insidious onset of bradykinesia, cognitive decline, and gait instability that began the year before our evaluation had been diagnosed with parkinsonian syndrome. Analysis of the cerebrospinal fluid showed lymphocytic pleocytosis, and a panel for PNS tested positive for anti-GAD65 and anti- CRMP5/CV2 antibodies. After investigation, a microcitoma was found in the lung. **Conclusions**: In light of our findings, we suggest considering PNS as an alternative diagnosis to parkinsonism-plus syndromes, in particular if bradykinetic syndrome is accompanied by other clinical manifestations including cognitive decline or ataxia in rapidly deteriorating patients. Earlier detection of PNS would lead to timelier identification of any occult tumors, therein promising improvement in the patient’s prognosis.

## 1. Introduction

Paraneoplastic neurological syndromes (PNSs) are a heterogeneous group of diseases affecting both the central (CNS) and peripheral nervous systems, triggered by an autoimmune response associated with the presence of a tumor. The diagnosis of PNSs can even precede the diagnosis of a neoplasm by many years. To date, several antineuronal antibodies have been identified and many of them are specifically found in some types of tumors [1]. The most frequent clinical manifestations of PNS include limbic encephalitis, paraneoplastic cerebellar degeneration, axonal sensory or sensorimotor polyneuropathy, opsoclonus–myoclonus, epileptic seizures, and encephalomyelitis. However, heterogeneous clinical phenotypes have also been described in the literature [1]. PNSs as etiologies of parkinsonian syndromes are rare, but some antibodies have been associated with these atypical syndromes: glutamic acid decarboxylase (GAD)-65 [2], Ma-2 [3], and CV2/collapsin response-mediator protein 5 (CRMP5) [4]. In this work, we present a case of insidious PNS, with double positivity for anti-GAD-65 and anti-CRMP5 antibodies, and characterized by cognitive and motor clinical manifestations, initially misdiagnosed as atypical parkinsonism. We also conducted a review of published PNS cases in patients presenting with parkinsonism; we focused on those cases with positivity for anti-GAD65 and anti-CRMP5 onconeural antibodies.

## 2. Clinical Scenario

### 2.1. Case Description

In May 2023, a 68-year-old Caucasian man with a recent history of cognitive decline, postural instability, and falls to the ground, some of them backwards, was seen at our center. The aforementioned patient’s symptoms started nearly one year before our evaluation, in June 2022. During this same period, the patient appeared less interested in performing activities he had always enjoyed, also showing executive hypofunction in a mixed condition of cognitive and social apathy. A progressive worsening of the executive and visual–spatial functions was noted by his wife, with his memory remaining intact. Motor involvement appeared nearly six months after cognitive impairment. The patient started being clumsy in his movements and had a bilateral tremor in his hands at rest. For these reasons, in February 2023, a parkinsonian syndrome was suspected by another neurologist, and the patient started a therapy with venlafaxine, selegeline, and Levodopa/Carbidopa. However, one month after commencing this therapy, it was suspended as the patient was afflicted with visual hallucinations. In April 2023, the patient underwent an F-18 fluorodeoxyglucose (18-FDG) positron emission tomography (PET) of his brain, which showed moderate fronto-temporo-parietal hypometabolism and mild hypometabolism in the right parietal region. In the same month, the patient underwent a brain magnetic resonance imaging (MRI) with gadolinium administration (gadobutrol, 0.1 mL/kg body weight), which revealed moderate to severe signs of cortical atrophy and ventricular enlargement. An I-123 ioflupane single-photon emission computed tomography (SPECT) (i.e., DaTSCAN™) showed dopaminergic deficiency in the left putamen.

### 2.2. Diagnostic Findings

**Figure 1:**
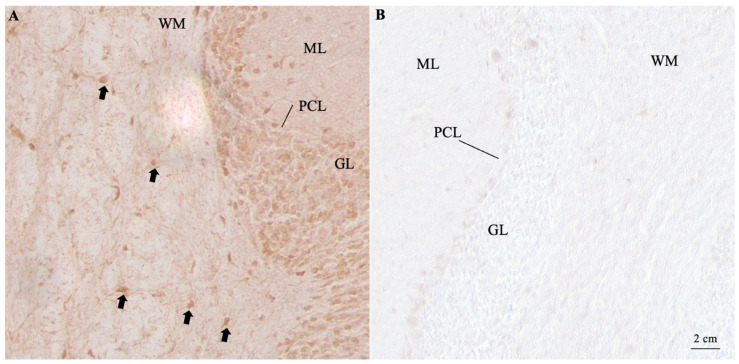
Patient serum showed two distinct bands corresponding to CV2/CRMP5 and GAD when assessed on a commercial line blot. When assessed using immunohistochemistry on paraphormaldehyde-fixed and perfused slices, the same serum (dilution 1:500) bound to oligodendrocytes within the white matter layer (WM) (black arrows), suggesting the presence of CRMP5/CV2 antibodies (**A**). An additional faint staining was observed in the granular layer (GL), possibly compatible with low-titer GAD antibodies (**B**). The scalebar is in the bottom right corner. ML = molecular layer; PCL = Purkinje Cell Layer.

In May 2023, the patient was admitted to the outpatient clinic for movement disorders at our hospital, where clinical examination showed slowing of saccadic eye movements, hypophonia, mild ataxia of walking and standing, bilateral bradykinetic syndrome with prevalence on the right side, plastic rigidity with trochlea of the wrists, in particular on the right side, mixed with mild bilateral spasticity. The osteotendinous reflexes were hyper-elicitable. Constructive apraxia was observed on the pentagon copy test. A parkinsonism-plus syndrome was suspected, and the patient had a diagnostic lumbar puncture, which revealed lymphocytic pleocytosis and increased protein concentrations: cerebrospinal fluid (CSF) lymphocytes were measured at 36 cells/mmc, proteins were measured at 1145 mg/dL, and glucose was measured at 67.6 mg/dL. An encephalitis process was suspected, and the patient was hospitalized. During hospitalization, investigations for viruses and bacteria in the CSF and blood tests yielded negative results. A double positivity for onconeural anti-GAD and anti-CRMP5/CV2 antibodies was detected by immunoblotting, while anti-surface antigens for NMDA-receptor, LGI1, GABA-B receptor, Casp-2, and AMPA1/2 tested negative when using EU90 fixed cell assay. Suspecting a paraneoplastic syndrome, a total body CT scan with a contrast medium was carried out. Despite some nodules in the lungs being detected, a PET scan of the chest ruled out a malignancy of the observed lesions. In September 2023, the antibody positivity were reanalyzed in another qualified laboratory by using part of the previously collected blood sample. In this case, the patient was tested for the presence of neuronal antibodies using indirect immunofluorescence on fixed monkey brain tissue (Euroimmun, Lubeck, Germany)—a method with high sensitivity [5]—which revealed a complex staining involving both the granular layer of the cerebellum and the oligodendrocyte cytoplasm. Confirmation tests showed positivity for both GAD antibodies (detected using an in-house fixed CBA) and CRMP5/CV2 antibodies (detected using a commercial immunoblot) (Figure 1). A neuropsychological evaluation in November 2023 revealed calculation difficulties, recall deficit, praxis-constructive disorder, working memory disorder, programming difficulties, and deficits in reasoning and conceptualization.

### 2.3. Treatment Performed

**Figure 2:**
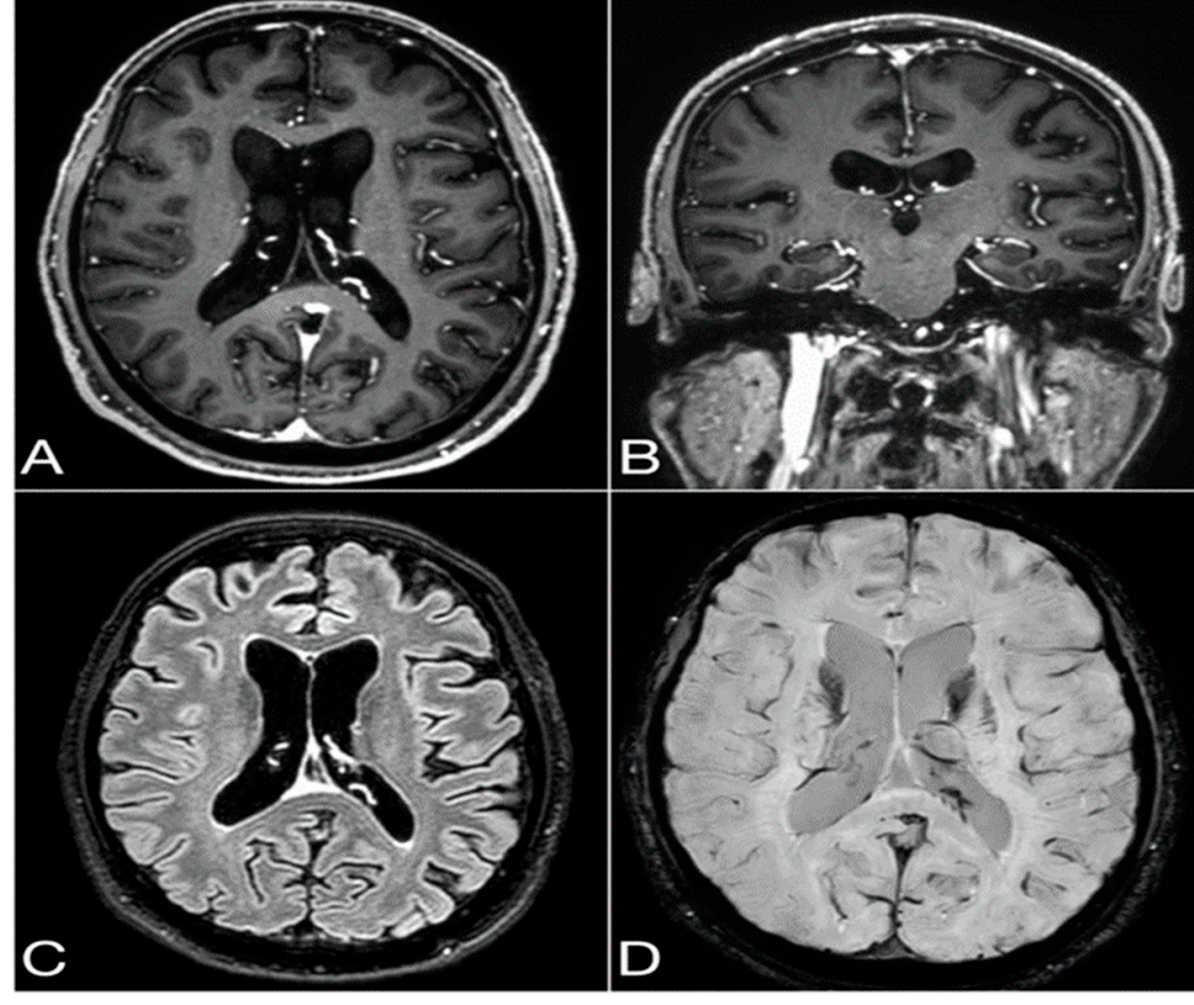
Last brain MRI performed with a 3T scanner (Philips Ingenia) with a 32-channel phased-array head coil in March 2024, showing moderate to severe signs of cortical atrophy and ventricular enlargement, with no sign of cerebral vasculopathy (neither leukoaraiosis nor microbleeds) or pathological post-contrast enhancements. Axial (**A**) and coronal (**B**) 1 mm thick multiplanar reconstructions from 3D T1-weighted Turbo Field Echo (TFE) with fat saturation and axial (**C**) 1 mm thick multiplanar reconstruction from 3D T2-weighted Fluid-Attenuated Inversion Recovery (FLAIR) with fat saturation (both sequences acquired after i.v. injection of gadolinium-based MRI contrast agent). Axial susceptibility-weighted imaging (SWI) processed magnitude image with a thickness of 2 mm (**D**).

Due to the fact that the patient had diabetes, we did not administer a high dose of methylprednisolone, but instead decided to start a treatment with intravenous immunoglobulins in November 2023, but it showed no improvement. In March 2024, the patient once again underwent a total body CT, a PET scan, and a brain MRI (Figure 2). The second CT scan showed an increase in the solid nodular formation size with irregular margins in correspondence with the apical segment of the right upper lobe, with a transverse diameter of approximately 10 mm vs. 6.5 mm. The PET scan showed accumulation of the metabolic tracer in the area of pulmonary parenchymal densification, with a nodular appearance, within the apical segment of the right upper lobe. A brain MRI was repeated in March 2024, which showed no significant changes in the previously described atrophy. A neoplastic pulmonary lesion in the apical region of the right upper lobe was suspected, so a further investigation via biopsy was carried out in April, showing small-cell lung carcinoma (SCLC). At the patient’s last visit in April 2024, a mild worsening of the bradykinesia and ataxia was observed.

## 3. Discussion

Here, we report on a case with dual positivity for anti-GAD65 and anti-CRMP5/CV2 antibodies in a patient with cognitive decline associated with parkinsonism and ataxia. Other cases of dual antineuronal antibodies have been reported in the literature. Although both anti-GAD65 and anti-CRMP5 antibodies are part of the group of antineuronal antibodies, no tumor was detected in our patient. However, the detection of one or more onconeural antibodies along with a neurological syndrome, even in the absence of tumor identification, according to the 2004 criteria [1], is sufficient for diagnosing a PNS. In particular, some onconeural antibodies are associated with a greater risk of tumors than others. In this regard, positivity for anti-Yo, anti-SOX1, anti-Hu, and anti-CRMP-5 antibodies confers the greatest risk of tumors [6]. It is important to highlight that, despite the great phenotypic variability of PNSs, some clinical manifestations are more typical than others. Notably, limbic encephalitis and cerebellar are typical features of anti-GAD65 encephalitis, while encephalomyelitis and sensory neuropathy are commonly associated with anti-CRMP-5 antibody-mediated PNS [6]. In our patient, the parkinsonian syndrome represents the main clinical feature. Parkinsonism is rare as a manifestation of PNS and only a few onconeural antibodies have been associated with it. In particular, anti-Ma2 and anti-Ri antibodies have demonstrated a correlation with parkinsonism in previous works [7]. Recently, reports have described cases of patients with parkinsonism and positivity for anti-CRMP-5 and anti-GAD-65 antibodies. Since parkinsonian syndrome is often complicated by ataxia and dysautonomia, these cases may have mimicked other diseases such as multiple system atrophy with prevalent parkinsonian features (MSA-P) and Lewy body disease. We report all the published cases of anti-CRMP5 and anti-GAD65 antibody-mediated PNS with a bradykinetic predominant parkinsonism currently in the literature in Table 1. Literature research was performed by searching for ‘’parkinsonism and CV2 antibodies’’, ‘’parkinsonism and CRMP5 antibodies’’, and ‘’parkinsonism and GAD antibodies’’.

Findings from our report and the few other case reports lead us to hypothesize that PNS with anti-CRMP5 and/or anti-GAD65 antibodies might mimic degenerative parkinsonism, in particular MSA-P or DLB, and this should be considered as a differential diagnosis for atypical parkinsonism, especially in cases with faster and insidious progression. Likewise, along with our patient, ten patients with parkinsonism and anti-CRMP5 and/or anti-GAD65 antibodies have been reported in the literature. Anti-CRMP5 has been more frequently reported than the anti-GAD65 antibody; the latter has only been identified in two male patients [13,14]. Excluding the three cases reported by Yu and his group due to lack of information, and excluding our case with dual antibodies, the symptom most frequently associated with anti-CRMP5 encephalitis, besides parkinsonism, has been ataxia, present in 4/6 (n = 67%) cases. Of these six patients, four were affected by small-cell lung cancer (SLCL), two were affected by breast cancer, and in one patient, no tumor was identified. SLCL was also the most frequent tumor in the ataxic subgroup. No tumor was found in the two cases of anti-GAD65 encephalitis. These cases of immune-mediated parkinsonism may present red flags, such as a faster disease progression—as in our case, which became evident in less than one year—and concomitant involvement of structures beyond the basal ganglia, such as the cerebellum and brainstem, as well as rapid cognitive decline. Moreover, since the pathogenetic mechanism underlying the clinical manifestations is an attack on the nervous system structures by antibodies, immunotherapies represent the appropriate treatments for these diseases. The reason why the two courses of intravenous immunoglobulins in our patient did not produce any beneficial effect could be the long time that elapsed between the onset and diagnosis of the disease, although the presence of antibodies with cytoplasmic neuronal antigens, unlike antibodies with cell-surface neuronal antigens, is not directly pathogenic, since it is considered an epiphenomenon of cytotoxic T cell damage. This explains the poor response to immunotherapy in people with PNS mediated by these types of antigens.

Therefore, in patients like ours, the most effective therapy could be direct chemotherapy and/or surgery [15,16].

The description of these subacute encephalitis—or, more appropriately, encephalopathies—might therefore be useful to clinicians for reducing diagnostic delays, consequently speeding up treatment initiation and optimizing therapeutic responses and clinical outcomes of patients.

## 4. Conclusions

This case underlines how the diagnosis of autoimmune encephalitis might be difficult, especially when the clinical course is not acute and is not complicated by red flags such as epileptic seizures. In fact, often, the involvement of multiple structures can lead to the composition of a varied clinical picture and raise other diagnostic suspicions. The different symptoms present in our patient reflect the antibody-mediated damage to various areas in which CRMP5 and GAD65 are normally expressed. In particular, the bradykinetic syndrome can be explained by the presence of CRMP5 in the basal ganglia, ataxia by the presence of GAD65 and CRMP5 in the cerebellum, apathy and executive difficulties by the expression of CRMP5 in the cingulate cortex and frontal regions, and cognitive decline by injuries to all the above-mentioned structures. In our case, to reach a correct diagnosis, it was first necessary to exclude the presence of atypical parkinsonism, suspected on the basis of the simultaneous presence of ataxia, bradykinetic parkinsonism, bilateral resting tremor, and cognitive decline. What made the case difficult was the fact that we observed the patient at approximately one year from the onset of symptoms, with the absence of a known tumor diagnosis preceding the onset of the neurological manifestations. As suggested by our literature research, the presence of rapidly progressing symptoms reflecting damage to multiple CNS structures requires the exclusion of other causes, especially a paraneoplastic origin such as lung tumors. In conclusion, we suggest excluding autoimmune encephalitis in cases of suspected atypical forms of parkinsonism with a subacute onset and fast progression of symptoms.

## Figures and Tables

**Table 1:** This table reports the cases present in the literature regarding anti-CRMP5 and anti-GAD65 antibody-mediated PNS characterized by parkinsonism.

N	Age	Sex	Antibody	Symptoms Associated with Parkinsonism	Tumor Found	References
1	46	F	CRMP5	Ataxia	SCLC	[8]
1	72	M	CRMP5	Ataxia, dysarthria, and constipation	SCLC	[8]
1	72	M	CRMP5	Urinary urgency and hoarseness of voice	SCLC	[9]
1	70	M	CRMP5	Ataxia, erectile dysfunction, severe dizziness while standing, hypotension, constipation, and urinary incontinence	No tumor found	[10]
1	52	F	CRMP5	Dizziness, speech disfluency, hypophonia, loss of taste, and occasional urinary incontinence	Breast cancer	[11]
3	NA	M2, F1	CRMP5	NA	NA	[12]
1	63	F	CRMP5	Gait and balance disturbances, pyramidal signs, dysarthria and inability to direct the gaze of the eyes downwards, slow saccades, and broken pursuits	Breast cancer	[4]
1	60	M	GAD65	Ataxic dysarthria, dysmetria of the left arm and leg, truncal stiffness and instability, and a broad-based ataxic gait	No tumor found	[13]
1	58	M	GAD65	Mild deficits in visuo-constructive and executive functions	No tumor found	[14]
1	70	M	CRMP5 and GAD65	Ataxia, hypophonia, and cognitive decline	SCLC	Our case report

## Data Availability

Data are available upon specific and reasonable request to the corresponding author.

## References

[1] Graus F., Delattre J.Y., Antoine J.E., Dalmau J., Giometto B., Grisold W., Honnorat J., Smitt P.S., Vedeler C., Verschuuren J.J.G.M. (2004). Recommended Diagnostic Criteria for Paraneoplastic Neurological Syndromes. J. Neurol. Neurosurg. Psychiatry.

[2] Pittock S.J., Yoshikawa H., Ahlskog J.E., Tisch S.H., Benarroch E.E., Kryzer T.J., Lennon V.A. (2006). Glutamic Acid Decarboxylase Autoimmunity with Brainstem, Extrapyramidal, and Spinal Cord Dysfunction. Mayo Clin. Proc..

[3] Dalmau J., Graus F., Villarejo A., Posner J.B., Blumenthal D., Thiessen B., Saiz A., Meneses P., Rosenfeld M.R. (2004). Clinical Analysis of Anti-Ma2-Associated Encephalitis. Brain A J. Neurol..

[4] Yu Z., Kryzer T.J., Griesmann G.E., Kim K.K., Benarroch E.E., Lennon V.A. (2001). CRMP-5 Neuronal Autoantibody: Marker of Lung Cancer and Thymoma-Related Autoimmunity. Ann. Neurol..

[5] Ricken G., Schwaiger C., De Simoni D., Pichler V., Lang J., Glatter S., Macher S., Rommer P.S., Scholze P., Kubista H. (2018). Detection Methods for Autoantibodies in Suspected Autoimmune Encephalitis. Front. Neurol..

[6] Graus F., Vogrig A., Muñiz-Castrillo S., Antoine J.C.G., Desestret V., Dubey D., Giometto B., Irani S.R., Joubert B., Leypoldt F. (2021). Updated Diagnostic Criteria for Paraneoplastic Neurologic Syndromes. Neurol. Neuroimmunol. Neuroinflamm..

[7] Xing F., Marsili L., Truong D.D. (2022). Parkinsonism in Viral, Paraneoplastic, and Autoimmune Diseases. J. Neurol. Sci..

[8] Purks J., Samkoff L. (2023). Rare Development of Anti-CRMP5 Parkinsonism and Worsening Ataxia While on Atezolizumab for SCLC: A Case Report (P4-5.031). Neurology.

[9] Tada S., Furuta M., Fukada K., Hirozawa D., Matsui M., Aoike F., Okuno T., Sawada J.I., Mochizuki H., Hazama T. (2016). Severe Parkinsonism Associated with Anti-CRMP5 Antibody-Positive Paraneoplastic Neurological Syndrome and Abnormal Signal Intensity in the Bilateral Basal Ganglia. J. Neurol. Neurosurg. Psychiatry.

[10] Yap S.M., Lynch T., MacMahon P., Murray B. (2017). Paraneoplastic Atypical Parkinsonism with Anti-CRMP5 Antibodies and Severe Caudate and Putaminal Hypometabolism on 18-Fluorodeoxyglucose Positron Emission Tomography of the Brain. Mov. Disord. Clin. Pract..

[11] Song J., Zhang Y., Lang Y., Wang Y.H., Shao J., Cui L. (2021). Parkinsonism and Dysautonomia with Anti-CV2/CRMP5 Associated Paraneoplastic Neurological Syndromes Mimicking Multiple System Atrophy: A Case Report. BMC Neurol..

[12] Wu X., Wang H., Xu G., Lin Y. (2019). Anti-CV2 Autoimmune Encephalitis with Parkinson-Like Symptoms and Bilateral Leukoencephalopathy—A Case Report. Front. Neurol..

[13] Nabil A., Houyam T., Adil B., Jawad O., Ahmed B. (2017). Severe Paraneoplastic Parkinsonism: A Rare Cause Revealing Breast Cancer. J. Clin. Neurol..

[14] Patel R.A., Joyce J., Witek N., Afshari M. (2021). GAD65 Antibody-Associated Neurologic Disease Presenting with Hemiparkinsonism at Onset. Neurol. Clin. Pract..

[15] Sadeghian H., Vernino S. (2010). Progress in the management of paraneoplastic neurological disorders. Ther. Adv. Neurol. Disord..

[16] Chiu D., Rhee J., Gonzalez Castro L.N. (2023). Diagnosis and Treatment of Paraneoplastic Neurologic Syndromes. Antibodies.

